# Semimetal–dielectric–metal metasurface for infrared camouflage with high-performance energy dissipation in non-atmospheric transparency window

**DOI:** 10.1515/nanoph-2024-0538

**Published:** 2025-01-17

**Authors:** Dongjie Zhou, Jinguo Zhang, Chong Tan, Liyan Li, Qianli Qiu, Zongkun Zhang, Yan Sun, Lei Zhou, Ning Dai, Junhao Chu, Jiaming Hao

**Affiliations:** State Key Laboratory of Infrared Physics, Shanghai Institute of Technical Physics, Chinese Academy of Sciences, Shanghai 200083, China; University of Chinese Academy of Sciences, No. 19A Yu Quan Road, Beijing 100049, China; 12478 Department of Materials Science and Institute of Optoelectronics, Shanghai Frontiers Science Research Base of Intelligent Optoelectronics and Perception, Fudan University, Shanghai 200433, China; Shanghai Key Laboratory of Metasurfaces for Light Manipulation, State Key Laboratory of Surface Physics, Key Laboratory of Micro and Nano Photonic Structures (Ministry of Education) and Department of Physics, 12478 Fudan University, 200433 Shanghai, China; Hangzhou Institute for Advanced Study, University of Chinese Academy of Sciences, Hangzhou 310024, China

**Keywords:** SMDM metasurface, infrared camouflage, broadband selective emission, deep-subwavelength

## Abstract

The development of novel camouflage technologies is of great significance, exerting an impact on both fundamental science and diverse military and civilian applications. Effective camouflage aims to reduce the recognizability of an object, making it to effortlessly blend with the environment. For infrared camouflage, it necessitates precise control over surface emissivity and temperature to ensure that the target blends effectively with the surrounding infrared background. This study presents a semimetal–dielectric–metal metasurface emitter engineered for the application of infrared camouflage. The metasurface, with a total thickness of only 545 nm, consists of a Bi micro-disk array and a continuous ZnS and Ti film beneath it. Unlike conventional metal-based metasurface design, our approach leverages the unique optical properties of Bi, achieving an average emissivity of 0.91 in the 5–8 μm non-atmospheric transparency window. Experimental results indicate that the metasurface emitter achieves lower radiation and actual temperatures compared to those observed in comparative experiments, highlighting its superior energy dissipation and thermal stability. The metasurface offers advantages such as structural simplicity, cost-effectiveness, angular insensitivity, and deep-subwavelength features, rendering it suitable for a range of applications including military camouflage and anti-counterfeiting, with potential for broad deployment in infrared technologies.

## 1 Introduction

The basic idea of camouflage is to minimize the detectable signature of an object and enable it to seamlessly blend with its surrounding background [1]. This approach is applicable to various electromagnetic wave bands [2], including the visible [3], [4], infrared [5], [6], and microwave regions [7], [8]. In contrast to visible and microwave bands, infrared surveillance systems typically work in a passive manner by detecting the infrared radiation emitted by objects [9]. Therefore, controlling of thermal radiation of targets plays a crucial role in infrared camouflage against infrared detection. Specifically, in order to achieve effective infrared camouflage for high temperature target, it is necessary for the device to exhibit low emission in specific atmospheric transparency windows (ATWs) [10], such as, 3–5 μm mid-wavelength infrared (MWIR) and 8–14 μm long-wavelength infrared (LWIR) ATW regions. At the meantime, it is normally required that the device should have high emission in non-atmospheric transparency window (non-ATW), such as 5–8 μm region, this would allow the device to reduce its temperature and further decrease the infrared radiation signal [11], [12], [13], [14], [15], [16], [17], [18], [19], [20].

So far, many types of artificially engineered materials have been investigated to developing wavelength-selective emitters for the applications of infrared camouflage based on the above strategy [21], [22], [23], [24], [25], [26], [27], [28], [29], [30], [31]. For example, photonic crystals based on dielectric–metal–dielectric (DMD) film perforated with a two-dimensional periodic aperture array have been exploited for high temperature infrared camouflage [32]. Metal–dielectric–metal (MDM) configuration metasurfaces made of metals such as gold (Au), silver (Ag), or aluminum (Al) have been proposed and demonstrated for infrared camouflage with thermal management [17], [18], [19]. Although these approaches have been successfully demonstrated to control the thermal emission of objects and realize impressive infrared camouflage effect, the performance, particularly in terms of energy dissipation efficiency in the non-ATW still could be improved.

In this study, we introduce a novel paradigm of semimetal–dielectric–metal (SMDM) metasurface emitter with high energy dissipation efficiency in the non-ATW for infrared camouflage. The SMDM metasurface consists of a layer of semimetal bismuth (Bi) micro-disk array, and a continuous titanium (Ti) film, separated by a zinc sulfide (ZnS) dielectric spacer. In contrast to previous works [33], [34], [35], [36], [37], [38], [39], semimetal Bi incorporated with Ti is employed instead of metals, such as, Au, Ag, and Al in our design. Semimetal Bi exhibits a unique optical response, which has negative values of the real part (*ε*_*real*_) of permittivity in the visible range [40], [41], similar to that of noble metals, while in the infrared region (see Section S1 in Supplementary Material for more detail), it possesses high positive *ε*_*real*_ values behavior as a lossy dielectric medium [42], [43]. By exploiting the overlapping of hybrid plasmonic resonances from the patterned Bi-based metasurface, the proposed metasurface emitter achieves an average emissivity of 0.91 in the 5–8 μm non-ATW, and exhibits a reduced emissivity of 0.72 in the MWIR ATW and 0.34 in the LWIR ATW, respectively. Experimental verification of the energy dissipation capability of the SMDM metasurface emitter revealed lower radiation and actual temperatures compared to samples lacking efficient emission capabilities at non-ATWs. Compared to a low emissivity metal film, the radiation temperature of the metasurface emitter is not much different from that the metal film, but the actual temperature is significantly lower, demonstrating its efficient energy dissipation capability and thermal stability. The proposed SMDM metasurface offers several advantages over previous designs, including a simpler structure, cost-efficiency, large-area fabrication, deep-subwavelength features, and angular insensitivity. These findings suggest promising applications that extend beyond infrared camouflage, including enhancements in thermal imaging [44], infrared anti-counterfeiting [45], and mid-infrared biological sensing [46].

## 2 Results and discussion

**Figure 1:**
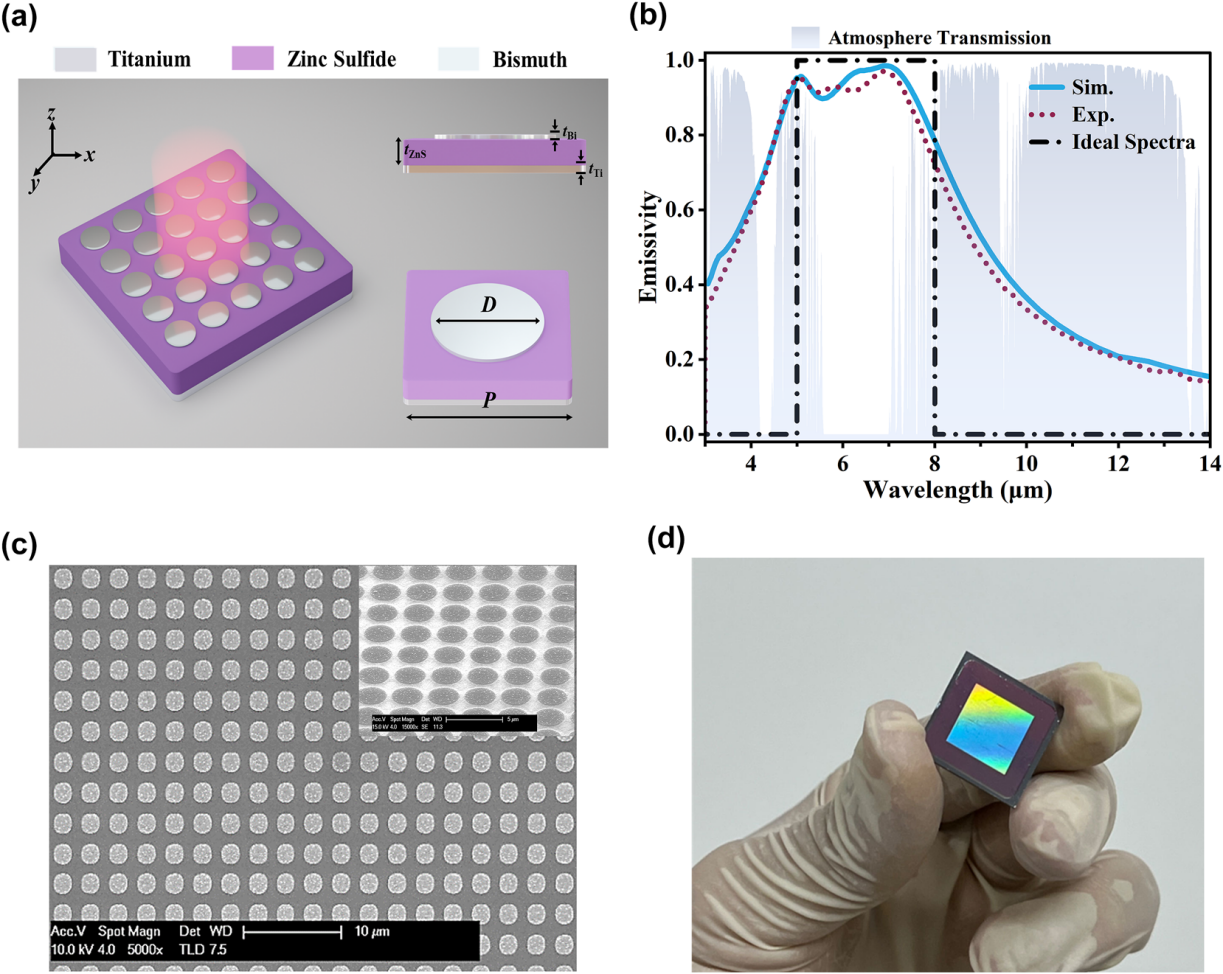
SMDM metasurface emitter for infrared camouflage concept and fabrication. (a) Illustration schematically depicting the proposed SMDM metasurface emitter. (b) Emissivity spectra for the metasurface emitter, where the simulation results (blue line) and experimental measurements (red dot line) are presented. These spectra were obtained with fixed parameters: *t*_*Bi*_ = 25 nm, *D* = 2.4 μm, *P* = 3.3 μm, *t*_*ZnS*_ = 400 nm and *t*_*Ti*_ = 120 nm. The comparison includes the ideal low emissivity infrared stealth spectra (black dash line) and the atmospheric transmission spectra (blue-shaded area). (c) Top-view and tilt-view SEM images of the experimental sample. (d) Optical images of the experimental sample.

Figure 1(a) illustrates schematic diagrams of the proposed SMDM metasurface selective emitter, which is basically comprised of three layers. The first layer is made of a periodic array of Bi micro-disks. *D* and *t*_*Bi*_ represent the diameter and the thickness of the Bi micro-disk, respectively, and *P* signifies the periodicity of the micro-disk array. The second and third layers are composed of ZnS and Ti films. Their thicknesses are denoted by *t*_*ZnS*_ and *t*_*Ti*_, respectively.

**Figure 2:**
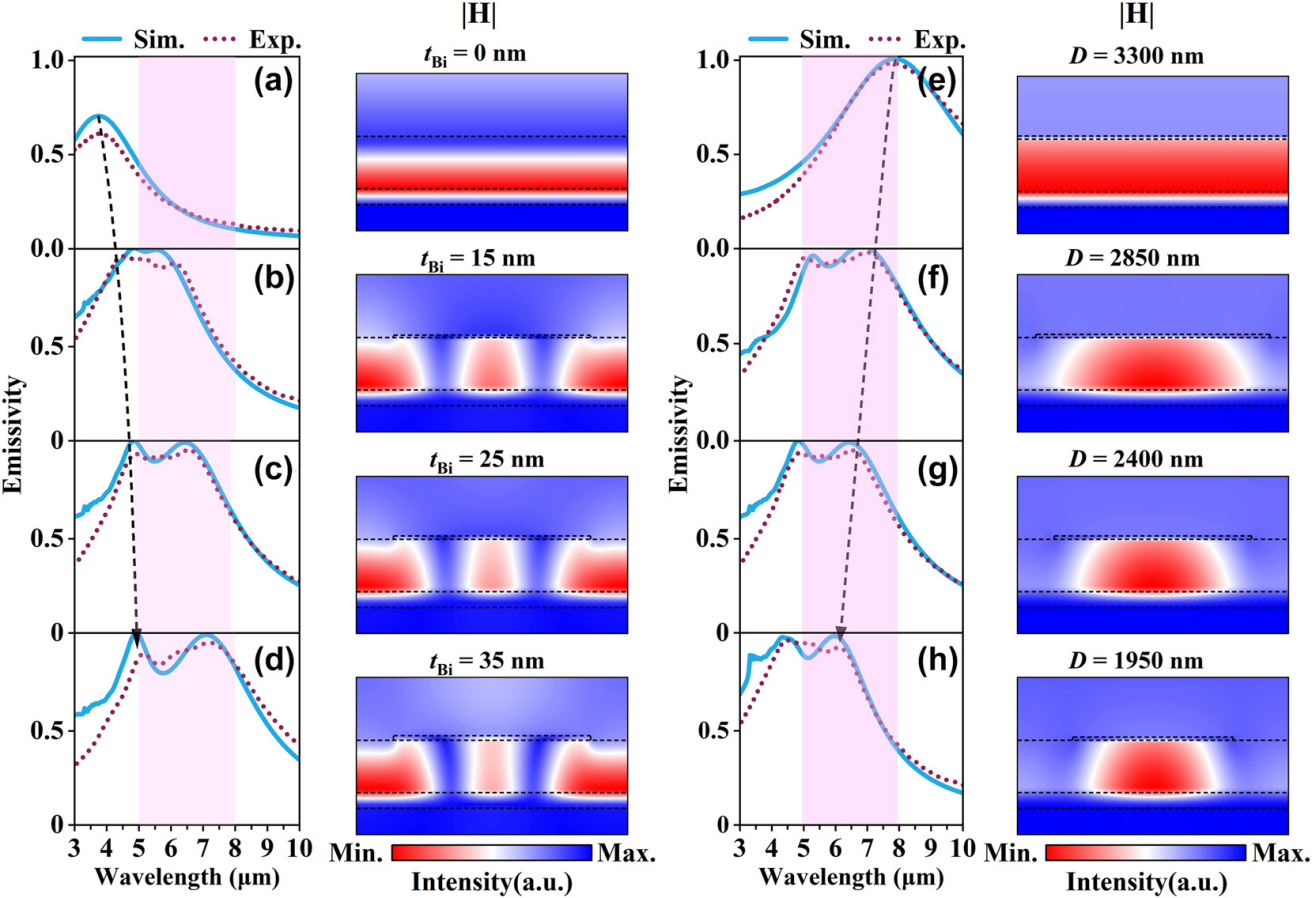
The relationship between the geometrical parameters of the Bi micro-disk with the optical response and resonance of SMDM emitter. (a–d) Comparison of simulated and experimental emissivity spectra for a series of SMDM metasurfaces with varying thicknesses of the Bi micro-disk (*t*_*Bi*_ = 0, 15, 25, and 35 nm), fixed *D* = 2.4 μm and accompanied by the magnetic field distribution of the resonant mode at shorter wavelengths. (e–h) Comparison of simulated and experimental emissivity spectra for a series of SMDM metasurfaces with varying diameters of the Bi micro-disk (*D* = 3,300, 2,850, 2,400, and 1,950 nm), fixed *t*_*Bi*_ = 25 nm and accompanied by the magnetic field distribution of the resonant mode at longer wavelengths. All results fixed the parameters as *t*_*ZnS*_ = 400 nm and *P* = 3.3 μm.

To determine the emission performance of the proposed emitter in the non-ATW, full-wave numerical simulations were conducted to investigate the dependence of emissivity on various geometrical factors. According to Kirchhoff’s law of thermal radiation, the emissive and absorptive properties of the structures are equal under thermal equilibrium. The emissivity (*E*) can be calculated as *E* = *A* = *1* − *R* − *T*, where *A*, *R*, and *T* represent the absorptance, reflectance, and transmittance of the proposed SMDM metasurface, respectively. The solid lines of Figure 2(a)–(d) show the simulated emissivity spectra for a series of Bi micro-disk thicknesses (*t*_*Bi*_ = 0, 15, 25, and 35 nm) at normal emission direction, with fixed other parameters as *D* = 2.4 μm, *P* = 3.3 μm, *t*_*ZnS*_ = 400 nm, and *t*_*Ti*_ = 120 nm. When the Bi micro-disk array is absent (*t*_*Bi*_ = 0 nm), the structure exhibits only one narrow resonant mode with the center wavelength of 3.9 μm located at MWIR ATW region. As the thickness of the Bi micro-disk (*t*_*Bi*_) increases, the center wavelength of resonant mode is redshifted to the non-ATW region, accompanied by the emergence of another emission resonance in the longer wavelength of this region. The solid lines of Figure 2(e) and (f) showcase the simulated emissivity spectra for four different diameters of the Bi micro-disk (*D* = 3.3, 2.85, 2.4, and 1.95 μm) at normal emission direction, with other parameters taken as *t*_*Bi*_ = 25 nm, *P* = 3.3 μm, *t*_*ZnS*_ = 400 nm, and *t*_*Ti*_ = 120 nm. When the top Bi micro-disk array becomes a continuous Bi thin film, namely, *D* = 3.3 μm, only one resonance emission peak located at the boundary between the LWIR ATW and non-ATW regions is observed as well. As the diameter *D* decreases, the longer wavelength emission peak gradually blueshifts and overlaps with that of the shorter wavelength, thus forming a wide emission band covering the range of the non-ATW. The relationships between the emission properties and other geometrical parameters are also investigated and presented in Figure S2 in Supplementary Material.

To understand the nature of the proposed SMDM structure, we simulated the electromagnetic field distributions for each emission peak wavelength and plotted the corresponding magnetic field distribution in the right column of each emission spectrum in Figure 2 (more details of field distributions are presented in Figure S3 of Supplementary Material). For the cases of *t*_*Bi*_ = 0 nm (see Figure 2(a)) and *D* = 3.3 μm (see Figure 2(e)), the structures composed of dielectric thin film(s) on top of a metallic reflector belong to typical Gires–Tournois resonator configuration [47], [48], [49], and the high emission/absorption peaks are attributed to the excitation of optical asymmetric Fabry–Perot–type interference resonance modes [50], [51], [52]. For the longer wavelength resonances, as shown in Figure 2(f)–(h), the magnetic field is strongly localized beneath the Bi micro-disk, it looks similar to the fundamental localized magnetic resonance supported by conventional MDM structure. For the shorter wavelength resonances (see Figure 2(b)–(d)), the magnetic field is concentrated not only under the Bi micro-disk but also in the area between two adjacent Bi micro-disks, the field profile exhibits the characterization of both fundamental and high-order plasmonic modes (more details of coupling of the cavity and micro-disk are presented in Section S4 of Supplementary Material). These results mean that such broadband emission/absorption originates from the contribution of hybrid multiplex plasmonic resonances.

To experimentally validate our idea, the proposed structures were fabricated using a combination of electron beam evaporation and laser lithography techniques (see Material and methods for more details of sample fabrication). The measured emissivity spectra of the fabricated emitters are correspondingly shown in Figure 2 as short-dotted lines. Good agreements are found by comparing the experimental spectra with the theoretical simulations. Figure 1(b) highlights the simulated and measured emission spectra of an SMDM metasurface emitter (denoted by “SMDM-S1”) with the optimal geometrical parameters of *t*_*Bi*_ = 25 nm, *D* = 2.4 μm, *P* = 3.3 μm, *t*_*ZnS*_ = 400 nm and *t*_*Ti*_ = 120 nm. The theoretical and experimental results show that the SMDM-S1 device exhibits an average emissivity of 0.91 within the non-ATW, 0.72 in the MWIR ATW and 0.34 in the LWIR ATW region. The morphology of the metasurface was characterized by a scanning electron microscope (SEM). As shown in Figure 1(c), the images show a uniformly periodic distribution of the Bi-based metasurface. Notably, there is some undulating surface topography on the Bi micro-disks, characterized by nanometer-level roughness introduced during the growth process. Figure 1(d) shows a photograph of the wafer-scale sample with micro-disk arrays, where the colored portion of the sample represents the structural region.

Table 1 describes the comparison of some recent research works in the field of infrared camouflage with thermal management. It notes that the proposed metasurface exhibits outstanding energy dissipation capability in the non-ATW region while maintaining excellent subwavelength thickness characteristics. This achievement is strongly related to the unique optical properties of Bi (with high refractive index and large absorption coefficient, more details are presented in Section S1 of Supplementary Material) and Ti (possessing strong optical absorption at the shorter wavelengths) materials [53] within the wavelength range of interest, which is challenging to achieve by using conventional metamaterials [14], [15], MDM metasurfaces [17], [18], [19], [20], [21], and promising that our device has great application potential in infrared camouflage. Furthermore, it’s important to note that while our proposed metasurface exhibits exceptionally high emissivity in the non-ATW region, its performance in the MWIR ATW region does not fully exceed that of previous studies. This limitation can be addressed by fine-tuning the structural parameters, as detailed in Figures S2 and S5 of the Supplementary Material. Moreover, since the proportion of mid-wave infrared radiation is relatively small at lower operating temperatures, our proposed SMDM device is suitable for LWIR camouflage applications at temperatures between 300 and 500 K. (see Section S5 in Supplementary Material for more details).

**Table 1:** Comparison of the performance of infrared camouflage with thermal management.

Ref.	Materials	Structure	Thickness	Thermal camouflage		Thermal Management
				*ɛ*_3–5 μm_	*ɛ*_8–14 μm_	*ɛ*_5–8 μm_
SMDM	Ti/ZnS/Bi	Metasurface	0.545 μm	0.72	0.34	0.91
[15]	Al_2_O_3_/AZO NPs	Metamaterials	1.2 μm	0.11	0.2	0.81
[17]	OPA/Cu	Metamaterials	1 μm	0.1	0.21	∼0.3
[18]	Au/GST/Si	Metasurface	1.25 μm	0.69	0.27	0.72
[19]	Au/ZnS/Au	Metasurface	0.6 μm	0.08	0.07	0.18
[20]	Al/SiO_2_/Al	Metasurface	0.62 μm	0.21	0.19	0.43
[21]	Au/SiO_2_/Au/PI	Metasurface	0.545 μm	0.14	0.08	0.31
[22]	Au/Polymide/Au	Metasurface	0.43 μm	0.19	0.07	0.42
[23]	Ag/Ge	4-layers MFS	1 μm	0.17	0.31	0.82
[24]	SiO_2_/Ge/ZnS/Pt/Au	8-layers MFS	4.4 μm	0.21	0.16	0.54
[25]	SiO_2_/TiO_2_/Ge	12-layers MFS	4.37 μm	0.14	0.21	0.86
[26]	Ge/ZnS	11-layers MFS	6.73 μm	0.11	0.12	0.61
[27]	Ge/YbF_3_	14-layers MFS	8.49 μm	0.06	0.01	0.68

**Figure 3:**
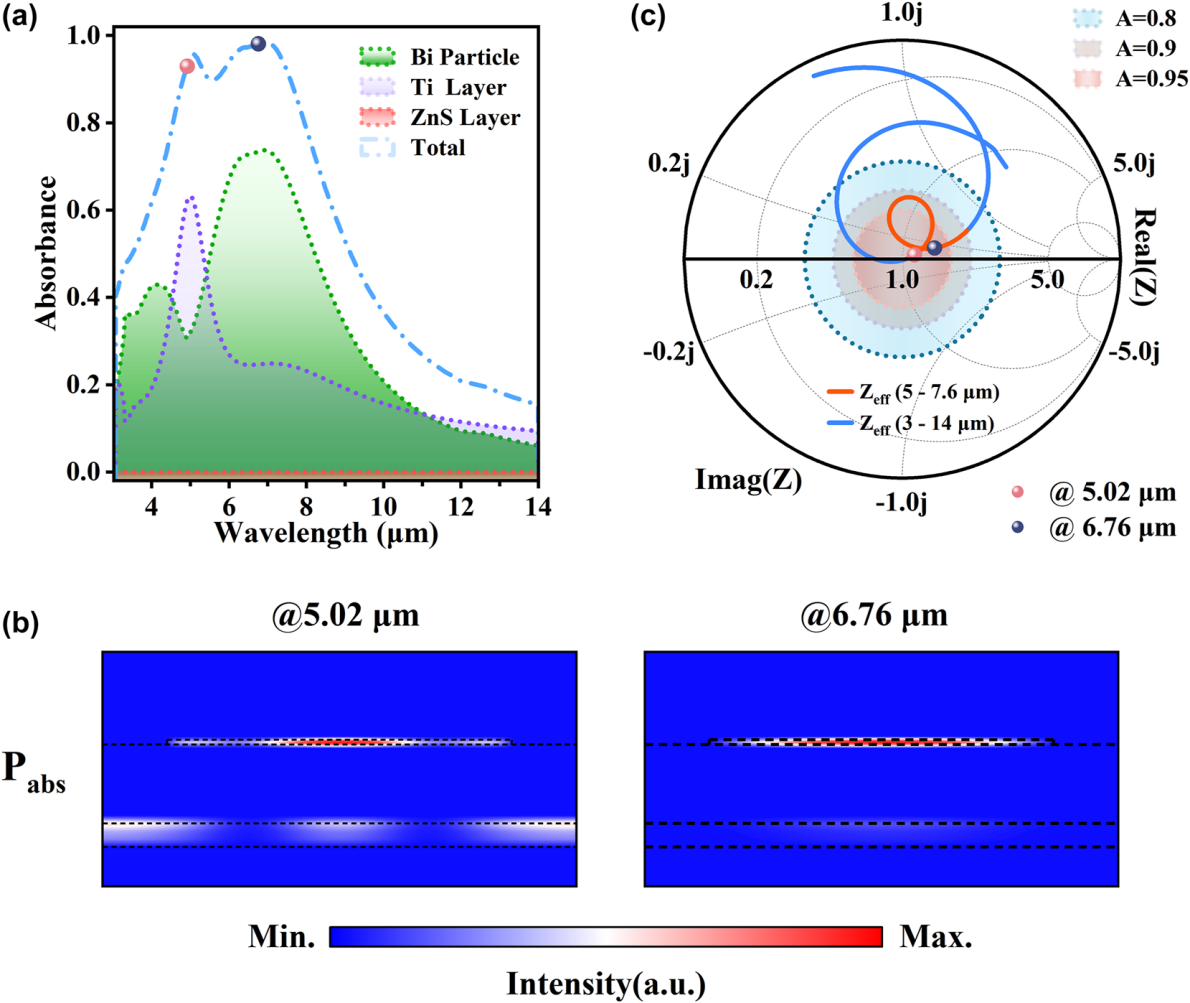
Power dissipation mechanism and optical impedance of the ‘SMDM-S1’ metasurface. (a) The normalized power loss spectra for the total and individual components of the SMDM-S1, with fixed thicknesses of Ti, ZnS, and Bi at 120 nm, 400 nm, and 25 nm, respectively, alongside a fixed period and diameter of the Bi micro-disk at 3.3 μm and 2.4 μm. (b) The normalized power loss density at the two resonant wavelengths of 5.02 μm and 6.76 μm. (c) The optical impedance (*Z*) of the SMDM-S1 metasurface across the wavelength range of 3–14 μm (blue line), with emphasis on the impedance where *A* > 0.9 (red line). The magenta, brown, and celeste short-dotted circles represent the impedances as *A* = 0.95, 0.9, and 0.85, respectively.

To provide further theoretical insights, additional numerical simulations and calculations were performed. Figure 3(a) shows the normalized power loss spectra for the total and each component of the SMDM-S1. In our structure, the energy dissipation contributions from the Ti layer and the Bi micro-disk vary across different wavelength ranges. Specifically, for shorter wavelengths of 5–5.4 μm, the Ti layer accounts for a larger portion of the energy dissipation, contributing 44 % of the total, while the Bi micro-disk contributes 37 %. In contrast, for the longer wavelength range of 5.4–8 μm, this trend reverses, with the Bi micro-disk dominating dissipation at 66 %, while the Ti layer contributes only 27 %. Overall, in the 5–8 μm range, the Bi micro-disk represents the largest share of energy dissipation at 62 %, compared to 30 % from the Ti layer. Figure 3(b) depicts the distributions of power loss density (W/m^2^) at the two emission peak wavelengths of 5.02 μm and 6.76 μm. As expected, the absorption at the longer wavelength is mostly due to the Bi micro-disk, while, at the shorter wavelength which is determined by both the Bi micro-disk and the Ti thin film. Moreover, we examined why the SMDM-S1 metasurface exhibits highly efficient absorption over the range of non-ATW. Given that the thickness of the 120 nm titanium (Ti) film in the bottom layer is sufficient to prevent light transmission and ensure that all energy dissipation occurs within the structure, the objective of maximizing absorption and emission translates into minimizing reflection. This relationship can be effectively analyzed through the optical impedance (*Z*). Figure 3(c) shows the optical impedance of the SMDM-S1 metasurface on a Smith impedance chart (see Section S6 in Supplementary Material for the details of the numerical computations). The center point of the chart corresponds to a device with *Z* = 1, indicating that the impedance of the device is ideally matched to the free space impedance, no incident light energy will be reflected *R* = 0 and *A* = 1. The magenta, brown, and celeste short-dotted circles represent the impedances as *A* = 0.95, 0.9, and 0.85, respectively. It is observed that the optical impedance of the SMDM-S1 metasurface falls within the circle of *A* = 0.9 over the wavelengths from 5 μm to 7.6 μm, the condition of impedance matching is reasonably well satisfied.

**Figure 4:**
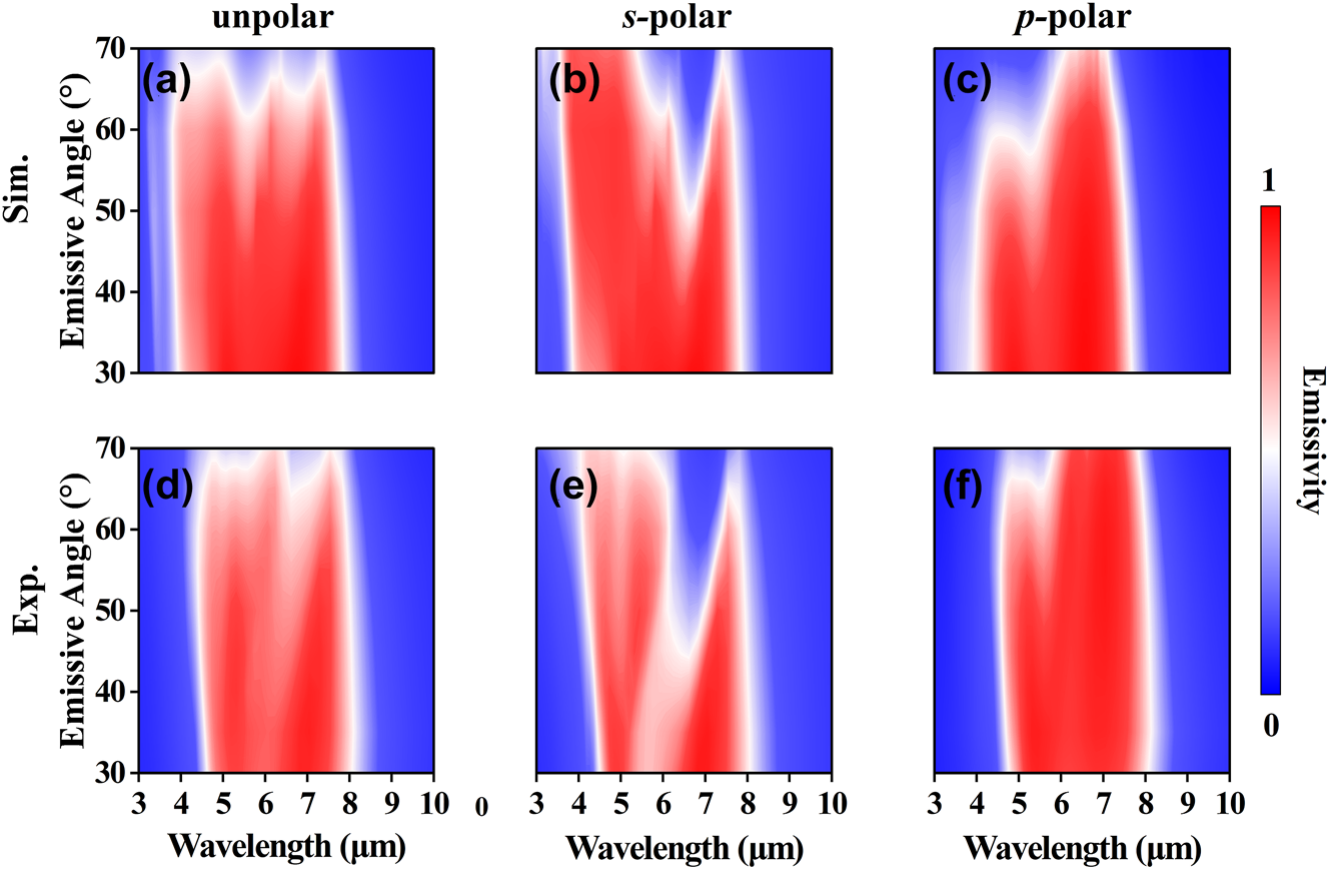
Angular dependences of optical properties of the proposed emitter. (a–c) Simulated and (e–f) experimentally measured emissivity spectra of the SMDM-S1 metasurface as functions of wavelength and emissive angle for unpolarized, *s*-polarized, and *p*-polarized light, respectively.

The angular dependence of infrared emission properties of the proposed metasurface was also investigated. Figure 4(a)–(c) displays the calculated emissivity (unpolarized, *s*-polarized, *p*-polarized) as a function of wavelength and emissive angle for the SMDM-S1 metasurface. The corresponding experimental results are shown in Figure 4(d)–(f). Both theoretical and experimental results show that the broadband selective emission is nearly independent of the emissive angle. The average emissivity in non-ATW remains 0.8 even at the emissive angle up to ∼60° (see Figure S7 in Supplementary Material). The feature endows the metasurface with omnidirectional energy dissipation ability in the non-ATW.

**Figure 5:**
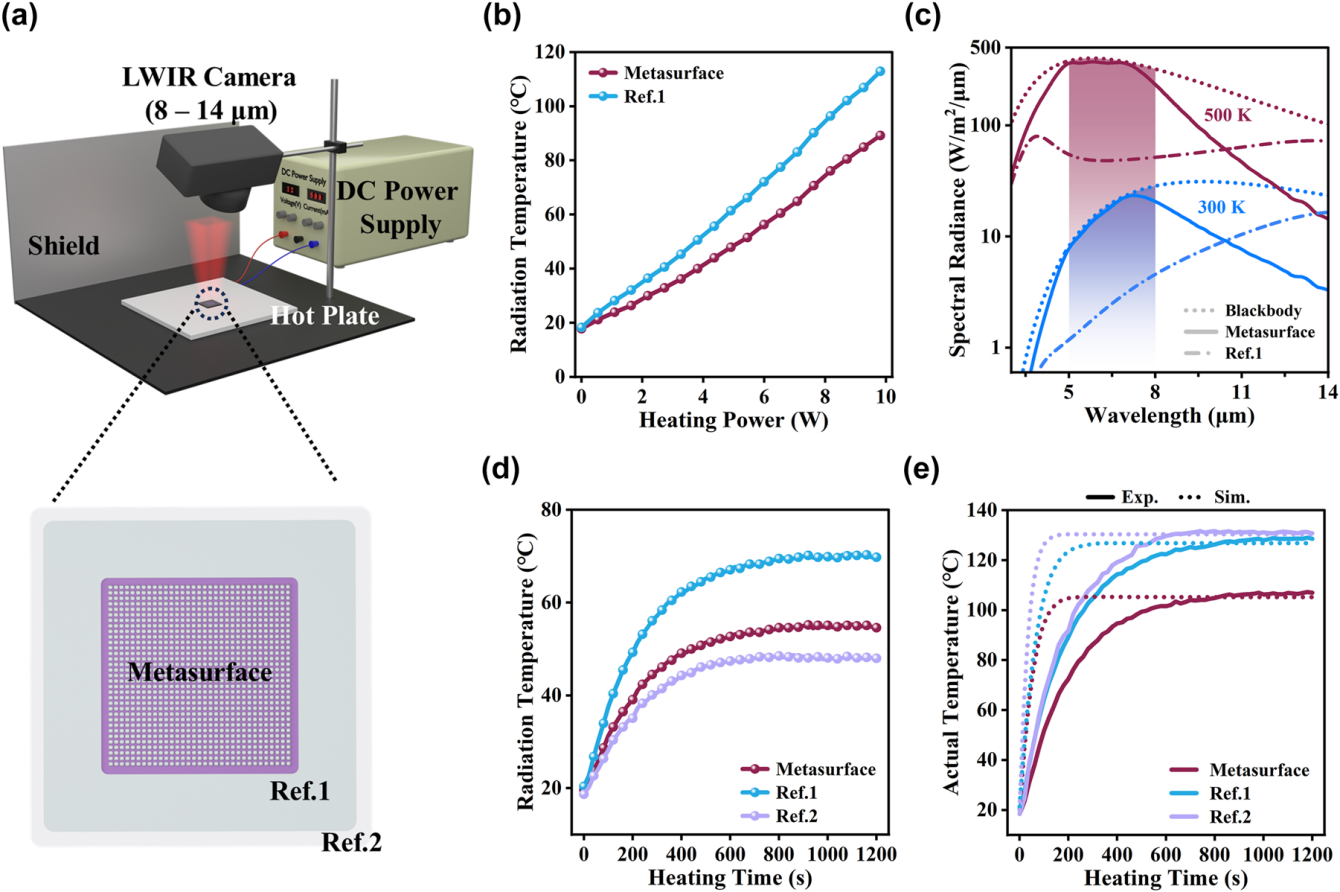
Experimental and simulation validation of the SMDM metasurface emitter thermal management performance. (a) Schematic illustration comparing the experimental setup and fabricated sample of the metasurface, Ref. 1, and Ref. 2. (b) Radiation temperature variation with heating power for the metasurface (reddish brown) and Ref. 1 (sky blue). (c) Spectral radiation of the metasurface (solid line), Ref. 1 (dash-dotted line), blackbody (dotted line), and ideal surface (shaded area) at 300 K (sky blue) and 500 K (reddish brown). (d) Average radiation temperatures of the metasurface, Ref. 1, and Ref. 2 (purplish grey) variation with heating time. (e) Average actual temperatures of the metasurface, Ref. 1, and Ref. 2 (purplish grey) variation with heating time both simulation (dotted line) and experiment (solid line).

To verify the advantages brought by the selective emission in non-ATW, thermal management performances of the proposed metasurface were evaluated under different situations. The bottom panel of Figure 5(a) illustrates a schematic of the SMDM-S1 metasurface selective emitter used for the thermal management evaluation, which is surrounded by two areas. The inner area (denoted by Ref. 1) consists of three layers of Bi, ZnS, and Ti films that has the same average emissivity in LWIR ATW (*E*_Ref._ _1: 8–14 μm_ = 0.34) as the SMDM-S1 but much lower emissivity in non-ATW (*E*_Ref._ _1: 5–8 μm_ = 0.14). The outer area (denoted by Ref. 2) is composed of only one layer of Ti film, and the emissivity of both LWIR ATW (*E*_Ref._ _2: 8–14 μm_ = 0.09) and non-ATW (*E*_Ref. 2: 5–8 μm_ = 0.11) is much lower than the SMDM-S1 metasurface (details of the reference samples are presented in Figure S8 in the Supplementary Material). The metasurface device was mounted on a hot plate which is electrically heated by a DC power supply system, as schematically shown in the top panel of Figure 5(a). Thermal infrared images were taken during the thermal measurement by an infrared camera with operating wavelength ranging from 8 μm to 14 μm. The emissivity of the camera was initially set to 0.95 (see Section S9 in the Supplementary Material for more information).

Figure 5(b) shows the radiation temperature results for the SMDM-S1 metasurface emitter and the control sample Ref. 1 under different heating powers. As can be observed, the radiation temperature of the metasurface emitter is always lower than that of the Ref. 1 control sample, and the temperature difference increases with the increase of heating power. At a heating power of 9.8 W, the temperature difference can be as high as ∼25 °C. This phenomenon is mainly attributed to the difference in energy dissipation capacity between the SMDM-S1 metasurface and Ref. 1 in the non-ATW region. Figure 5(c) shows the spectral radiance of the SMDM-S1 metasurface and Ref. 1 at temperatures of 300 K and 500 K. For comparison, the spectral radiance of the blackbody is also plotted in Figure 5(c). At 300 K, the radiation power densities of the metasurface and the reference sample Ref. 1 within the 8–14 μm LWIR ATW region are 57.61 W/m^2^ and 60.1 W/m^2^; for the 5–8 μm non-ATW, the radiation power densities of the metasurface and Ref. 1 are 52.97 W/m^2^ and 7.91 W/m^2^, respectively. At 500 K, the metasurface and the reference sample Ref. 1 have radiation power densities of 400.08 W/m^2^ and 383.69 W/m^2^ respectively in the LWIR ATW, and 1,038.03 W/m^2^ and 149.82 W/m^2^ respectively in the non-ATW. It notes that the radiative capacity of the metasurface is very close to that of Ref. 1 in the LWIR ATW, however, in the non-ATW, the radiation capacity of the metasurface is much stronger than that of the reference sample. Radiative cooling induced by such strong energy dissipation leads to a lower temperature of the metasurface than the reference sample. Moreover, with the increase of temperature, the greater the difference in radiation power between the metasurface and Ref. 1, this is why the radiation temperature difference between the two becomes larger as the heating power increases.

**Figure 6:**
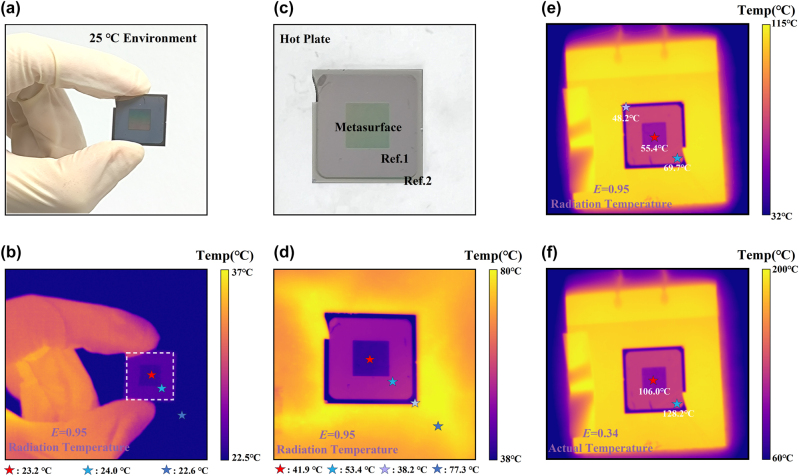
Optical image and IR radiation temperature distribution (metasurface: red, Ref. 1: sky blue, Ref. 2: purplish grey, background: blue). (a–b) The hand-held samples at room temperature represented the initial state. (c–d) Intermediate state. (e) Steady-state temperature distribution setting the in-camera emissivity to *E* = 0.95. (f) Steady-state temperature distribution setting the in-camera emissivity to *E* = 0.34.

Figure 5(d) shows the radiation temperature results of the SMDM-S1 metasurface emitter and the control samples Ref. 1 and Ref. 2 according to heating time for a fixed heating power of 6 W. Figure 6(a)–(e) display the infrared images (captured by the infrared camera with the emissivity of 0.95) and the corresponding photographs of this measurement at different temperature stages (6(a, b): initial state (room temperature); 6(c, d): intermediate state temperature; 6(e): steady state temperature). The radiation temperature values are retrieved at the positions labeled by the stars in Figure 6(b), (d), (e). The solid lines of Figure 5(e) show the actual temperature results of the SMDM-S1 metasurface emitter and the reference samples Ref. 1 and Ref. 2 according to heating time for a fixed heating power of 6 W, which are obtained by changing the infrared camera emissivity to 0.34 for the metasurface emitter and Ref. 1 (see Figure 6(f)), and 0.09 for the sample Ref. 2 (see Figure S10 in Supplementary Material). To confirm these results, heat transfer numerical simulations were performed, and the corresponding results are presented in Figure 5(e) as short-dotted lines (see Section S11 in Supplementary Material for more details). The theoretical results for the steady state temperature of the three cases are in good agreement with the experimental values. As shown in Figure 5(d) and (e), both the radiation temperature and actual temperature of Ref. 1, as expected, are higher than the metasurface emitter. Although Ref. 2 exhibits a lower radiation temperature than the metasurface emitter, its actual temperature (130.7 °C) is much higher than of the metasurface emitter (106.0 °C) at the steady state, this can yield thermal instability of the system. In other words, the proposed metasurface emitter can not only be utilized for camouflage by reducing the infrared signature in the ATW but also offers better thermal stability to the target due to its superior radiative cooling capacity through energy dissipation in the non-ATW.

## 3 Conclusions

In conclusion, we have developed an SMDM metasurface emitter, which is engineered to achieve high energy dissipation efficiency in the non-ATW, making it ideal for advanced infrared camouflage applications. Our SMDM metasurface utilizes semimetal Bi, which displays high positive values in the real part of the dielectric constant in the IR range, acting as a lossy dielectric medium. This configuration enables hybrid plasmonic resonances, resulting in effective broadband emission in the 5–8 μm non-ATW. Comparative experiments show that our metasurface can achieve up to a 25 °C reduction in radiative temperature compared to a sample lacking non-ATW energy dissipation capability (Ref. 1), highlighting its superior energy dissipation performance. Additionally, while the radiation temperature of our metasurface is slightly higher than a metal film (Ref. 2), it maintains a significantly lower actual temperature, demonstrating its good thermal stability. These findings offer a straightforward method for achieving broadband, high-efficiency emission in the non-ATW through strategic structural design, which benefits IR camouflage applications and suggests potential for wide deployment in IR technologies.

## 4 Materials and methods

### 4.1 Optical constants measurement

The optical constants of the deposited Bi film were measured and fitted in the infrared range using infrared spectroscopic ellipsometry (Sendira Sentech, 1,500–25,000 nm). This measurement accounts for a roughness layer of approximately 7 nm, while the optical constants of Ti and ZnS were obtained from the literature [53].

### 4.2 Electromagnetic simulation

The finite-difference time-domain (FDTD) method was utilized to simulate the reflectance and absorbance spectra and the electromagnetic field information inside the structure. The S-parameters were calculated by rigorous coupled wave analysis (RCWA).

### 4.3 Device fabrication

The SMDM metasurface emitter, Ref. 1, and Ref. 2 were prepared on the same wafer using electron-beam evaporation (Syskey Technology UHEB-LC6-03 system). Ti and ZnS were deposited at room temperature, with deposition rates of 2 A/s and 10 A/s, respectively. The metasurface array was patterned using laser lithography (SVG Microlab) with UV photoresist (AZ5214). A 25 nm layer of Bi was deposited at a rate of 2 A/s. After lift-off, an additional 153 nm thick layer of Bi was deposited, properly aligning Ref. 1 around the metasurface array.

### 4.4 Sample characterization

The micro-scale Fourier transform infrared spectrometer (Thermo Scientific Nicolet iN10) was used to obtain the different sizes of micro-area metasurface array reflectance spectra. The angle-resolved reflectance spectra over a range of 30°–70° were obtained using a Fourier transform infrared spectrometer (Thermo Scientific Nicolet iS50), both covering a spectral range of 8,000–400 cm^−1^. Additionally, the morphologies of the SMDM metasurface were characterized using a scanning electron microscope (FEI Sirion 200) with an accelerating voltage of 10 kV. Thermal measurements were conducted using an LWIR camera (Fotric 220) with a default emissivity of 0.95, operating in the 8–14 μm wavelength range. The AnalyzIR software package was used to extract radiation and actual temperature curves from LWIR images for the center (SMDM-S1) and peripheral (Ref. 1, Ref. 2) areas of the device. Heating power ranging from 0 to 10 W were applied using a DC power supply linked with a ceramic hotplate. The temperature trends over time for SMDM-S1, Ref. 1, and Ref. 2 were recorded using a 6 W heating power and the steady-state temperature of the hotplate is ∼135 °C measured by a thermocouple.

## Supplementary Material

Supplementary Material Details
