# Supplementary material for: Semimetal–dielectric–metal metasurface for infrared camouflage with high-performance energy dissipation in non-atmospheric transparency window
Source: Nanophotonics. 2025 Jan 17;14(8):1101–11. doi: 10.1515/nanoph-2024-0538 (PMC12019945; doi:10.1515/nanoph-2024-0538)
Supplement: Supplementary file 1 — Supplementary Material Details [file j_nanoph-2024-0538_suppl_001.docx]

**Supplementary Material**

**Semimetal-dielectric-metal metasurface for infrared camouflage with high-performance energy dissipation in non-atmospheric transparency window**

Dongjie Zhou^1, 2^, Jinguo Zhang^3^, Chong Tan^1, 2^, Liyan Li^3, 4^, Qianli Qiu^1, 2^, Zongkun Zhang^1, 2^, Yan Sun^1, 2, *^, Lei Zhou^4^, Ning Dai^1, 5^, Junhao Chu^1, 3^, and Jiaming Hao^3, *^

^1^ State Key Laboratory of Infrared Physics, Shanghai Institute of Technical Physics, Chinese Academy of Science, Shanghai 200083, China

^2^ University of Chinese Academy of Sciences, No. 19A Yu Quan Road, Beijing 100049, China

^3^ Department of Materials Science & Institute of Optoelectronics, Shanghai Frontiers Science Research Base of Intelligent Optoelectronics and Perception, Fudan University, Shanghai 200433, China

^4^ Shanghai Key Laboratory of Metasurfaces for Light Manipulation, State Key Laboratory of Surface Physics, Key Laboratory of Micro and Nano Photonic Structures (Ministry of Education) and Department of Physics, Fudan University,

200433 Shanghai

^5^ Hangzhou Institute for Advanced Study, University of Chinese Academy of Sciences, Hangzhou 310024, China

*Corresponding author: [sunny@mail.sitp.ac.cn](mailto:sunny@mail.sitp.ac.cn); [jmhao@fudan.edu.cn](mailto:jmhao@fudan.edu.cn)

**Contents**

[**Section S1 Bismuth Infrared Optical Properties 3**](#_Toc183349338)

[**Section S2 Impact of the geometric parameters of metasurface on its emission property. 4**](#_Toc183349339)

[**Section S3 The application situation for ‘SMDM-S1’ and the design of the ‘SMDM – MWIR’ 5**](#_Toc183349340)

[**Section S4 Electric field distributions for the metasurface with different geometrical parameters. 7**](#_Toc183349341)

[**Section S5 Limitation condition of Bi Micro-disk 8**](#_Toc183349342)

[**Section S6 Effective metasurface impedance extraction 9**](#_Toc183349343)

[**Section S7 Average emissivity simulation and experiment of the sample in non-ATW. 10**](#_Toc183349344)

[**Section S8 Spectral radiance, atmospheric radiance power, solar radiance power, average emissivity calculation and reference sample design. 11**](#_Toc183349345)

[**Section S9 A photograph of experimental setup for thermal management. 14**](#_Toc183349346)

[**Section S10 An infrared Image captured by the infrared camera with the emissivity of E=0.09 15**](#_Toc183349347)

[**Section S11 Numerical calculation of temperature difference between metasurface, Ref.1, and Ref.2. 16**](#_Toc183349348)

**Section S1 Bismuth Infrared Optical Properties**


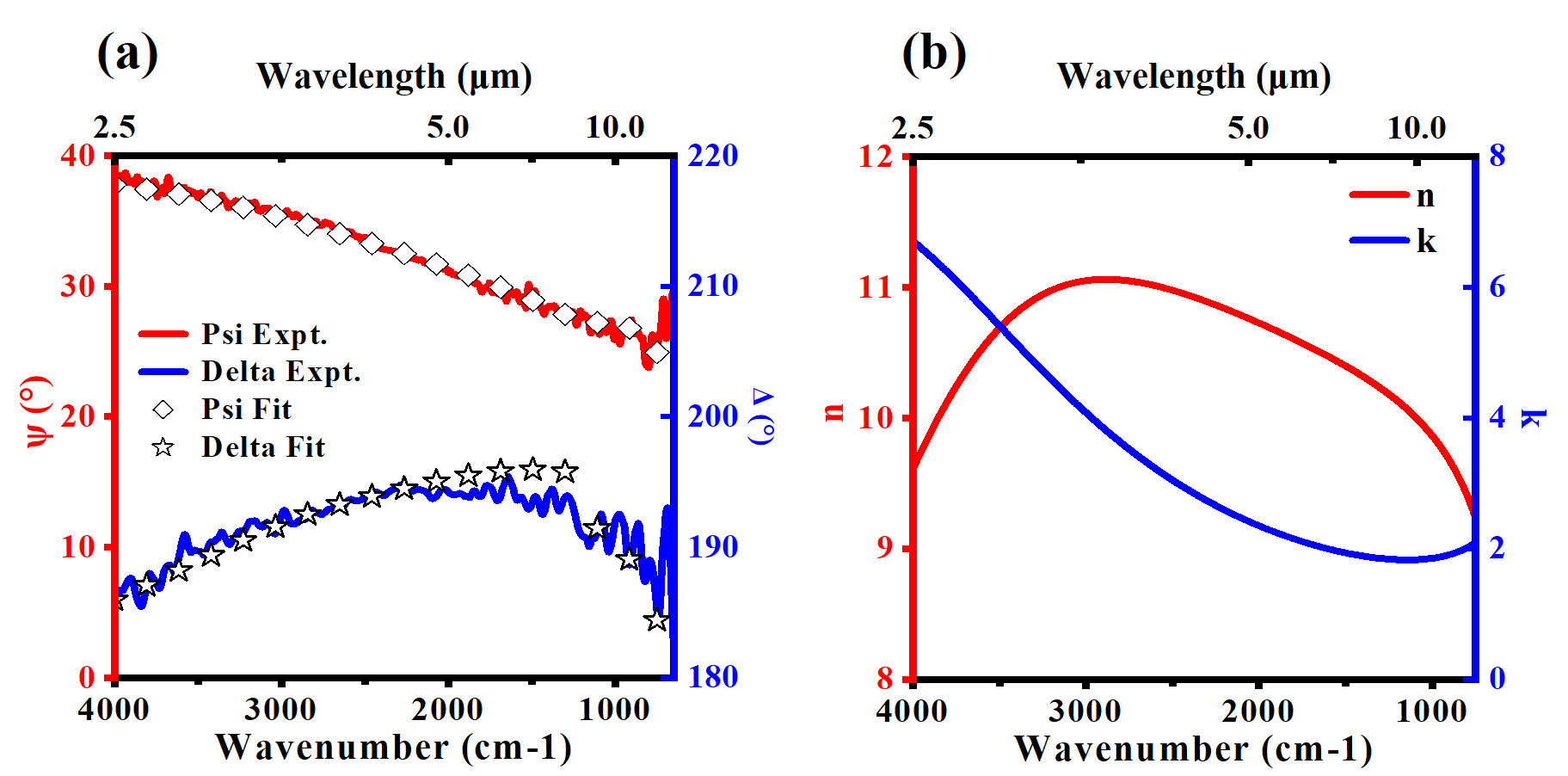


**Figure S1.** (a) Experimental measured *Ψ* and *Δ* (symbol curves) for Bi thin-film, and the fitted results from the Drude–Lorentz multiple oscillators model (solid curves). (b) Real and imaginary parts of refractive index for the Bi thin-film retrieved from the infrared ellipsometry data analysis.

Section S2 Impact of the geometric parameters of metasurface on its emission property.

To explore the influence of geometrical parameters on the emission spectra of the SMDM metasurface, an extensive investigation was undertaken, surpassing the findings delineated in Figure 2 of the primary text. Employing the FDTD method with predefined optimal structure parameters (*t_ZnS_* = 400 nm, *t_Ti_* = 120 nm, *t_Bi_* = 25 nm, *D* = 2400 nm, and *P* = 3300 nm), meticulous scrutiny was conducted, with specific emphasis on *t_ZnS_*, *t_Bi_*, and *D*. The outcomes elucidate that augmenting the thickness of *t_ZnS_* engenders a discernible redshift in the spectra, as depicted in Figure S2(a). Meanwhile, emission peaks proximal to shorter wavelengths evince minimal variation with increasing *t_Bi_*, whereas those associated with longer wavelengths undergo a redshift, as evidenced in Figure S2(b). Furthermore, expanding the diameter of the micro-disk precipitates a shift towards longer wavelengths, concomitant with the disappearance of emission peaks linked to shorter wavelengths, particularly evident as *D* converges towards the period, as delineated in Figure S2(c).


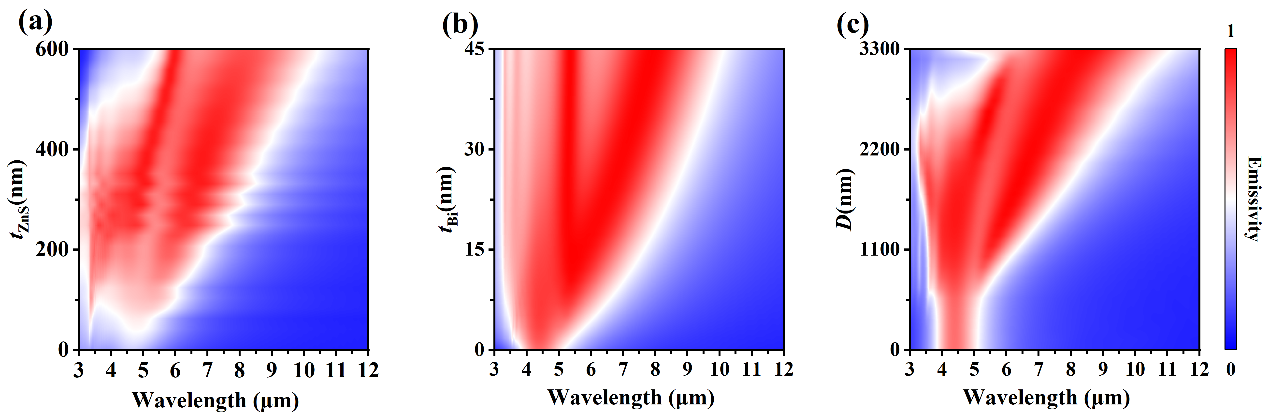


**Figure S2.** (a) Simulated emission spectra as a function of wavelengths and ZnS spacer thickness (*t_ZnS_*). (b) Simulated emission spectra as a function of wavelengths and Bi micro-disk thickness (*t_Bi_*). (c) Simulated emission spectra as a function of wavelengths and Bi micro-disk diameter (*D*).

Section S3 Electric field distributions for the metasurface with different geometrical parameters.


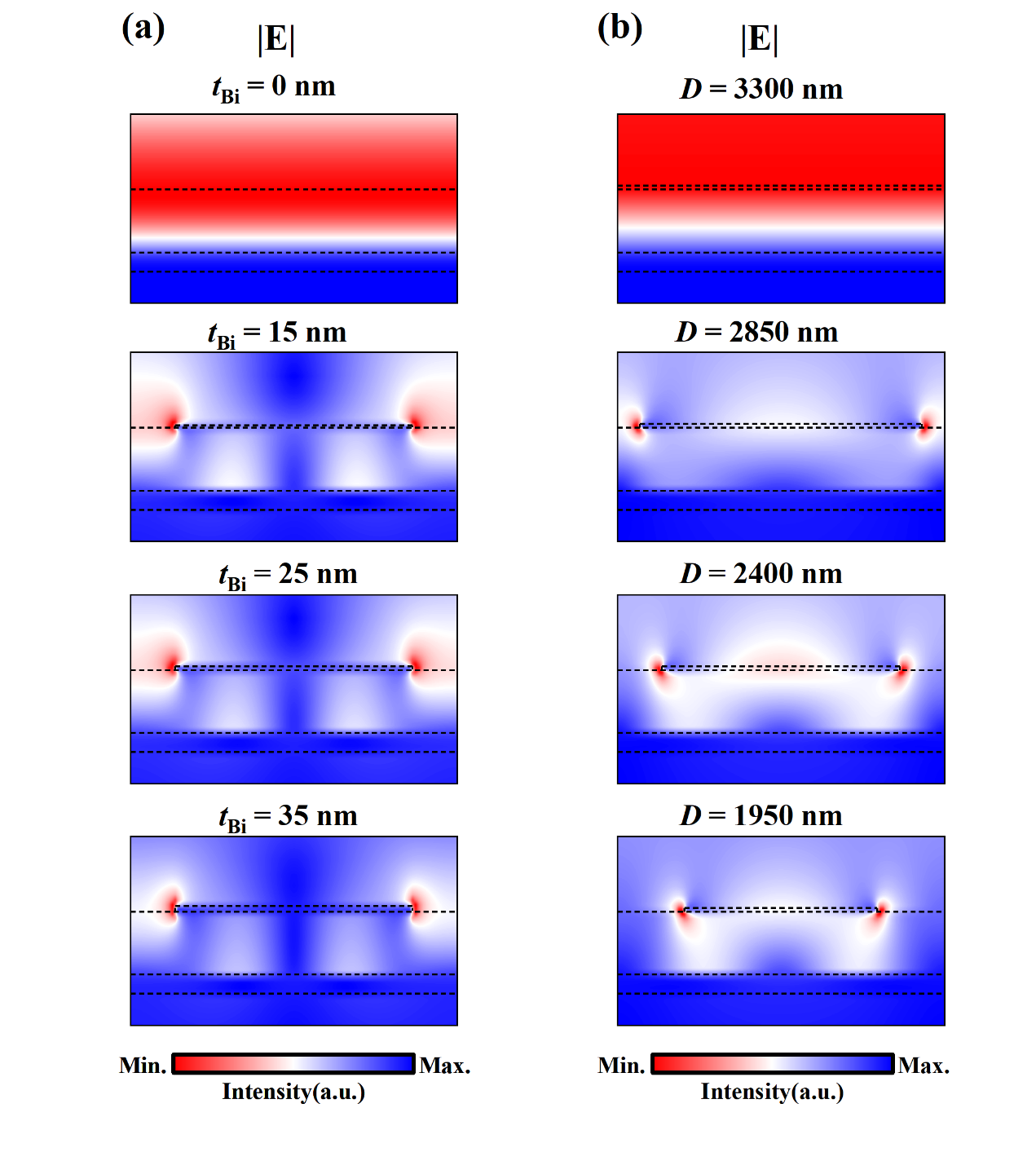


**Figure S3.** All results fixed the parameters as *t_ZnS_* = 400 nm and *P*=3.3μm. (a) The electric field distribution of the resonant mode at shorter wavelengths for a series of SMDM metasurfaces with varying thicknesses of the Bi micro-disk (*t_Bi_* = 0, 15, 25, and 35 nm), fixed *D* =2.4μm. (b) The electric field distribution of the resonant mode at longer wavelengths for a series of SMDM metasurfaces with varying diameters of the Bi micro-disk (*D* = 3300, 2850, 2400, and 1950 nm), fixed *t_Bi_* =25 nm.

Section S4 Limitation condition of Bi Micro-disk

To elucidate the coupling between the plasmonic structure and the cavity mode, two limiting cases were examined by calculating the spectrum and electromagnetic field distribution of the metasurface (D = 100 nm and D = 3200 nm). As illustrated in Fig. S5, when the diameter of the Bi disk is small, a prominent resonance peak at 4.2 μm is excited, with the magnetic field distribution at this peak clearly demonstrating the coupling between the plasmonic structure and the GT cavity of ZnS-Ti. Conversely, for the larger disk (D = 3200 nm), the resonance at shorter wavelengths nearly disappears, resulting in a spectrum like that of the Bi-ZnS-Ti (MDM planar structure) shown in Fig. 3. At the resonance peak of 7.8 μm, the plasmonic structure effectively couples with the FP resonance generated by Bi-ZnS-Ti.


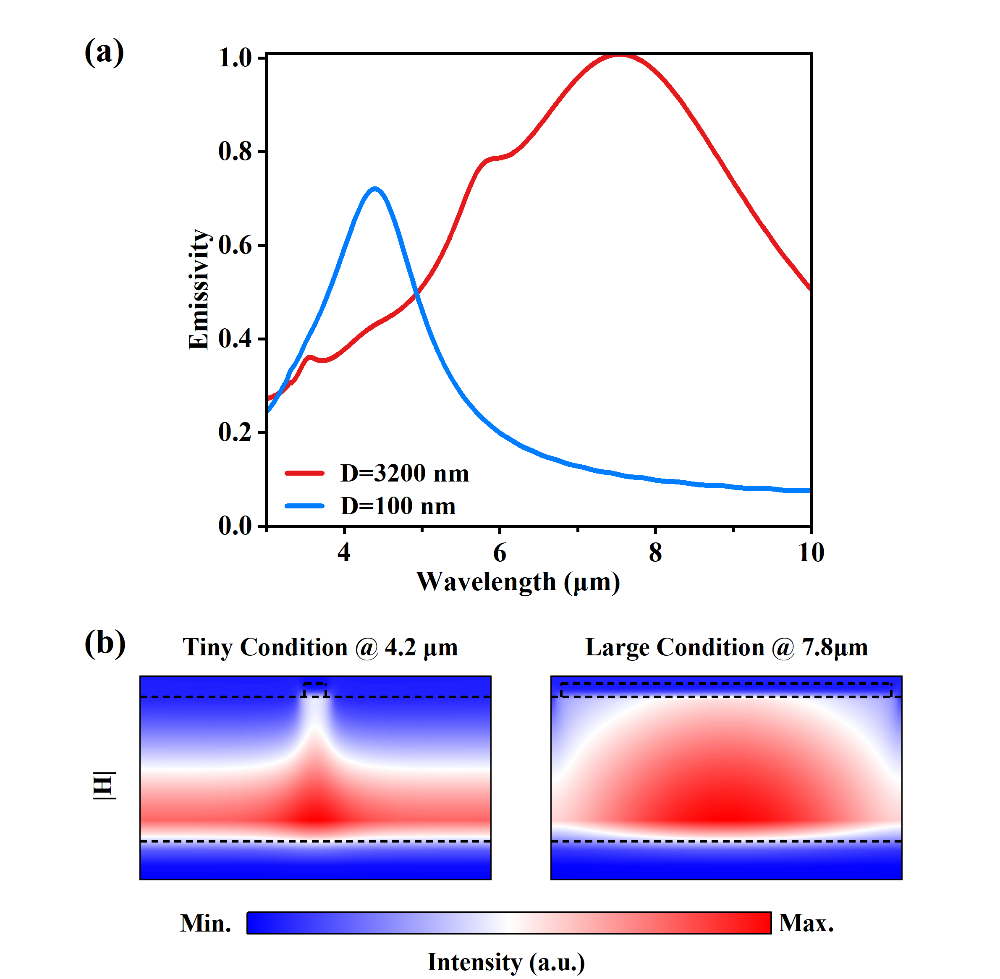


**Figure S4** (a) The simulated emissivity spectrum of the tiny disk (*t_Ti_* =120nm, *t_ZnS_* =400nm, *t_Bi_*=25nm, *D* =100 nm, and *P*=3300 nm) and the large disk (*t_Ti_* =120nm, *t_ZnS_* =400nm, *t_Bi_*=25nm, *D* =3200 nm, and *P*=3300 nm). (b) The simulated magnetic field distribution of the tiny disk and the large disk.

Section S5 The application situation for ‘SMDM-S1’ and the design of the ‘SMDM – MWIR’

While the primary text highlights the superior energy dissipation of the ‘SMDM-S1’ structure within the 5–8 μm range, we acknowledge that its performance in the 3–5 μm (MWIR) band remains suboptimal. However, potential optimizations have been identified to enhance its effectiveness within this range. According to Planck’s law, the distribution of blackbody radiation varies with temperature. As shown in Table S5, which presents the radiation ratio for different wavebands normalized to the total blackbody radiation power in the 3–14 μm range, the proportion of radiation in the 3–5 μm band is relatively low for temperatures between 300-500 K. Consequently, the average emissivity of the ‘SMDM-S1’ device across the entire atmospheric transmission window (ATW), which includes both the 3–5 μm and 8–14 μm bands, is 0.35, 0.39, and 0.45 at 300 K, 400 K, and 500 K, respectively. Given the relatively suboptimal radiative properties in the 3–5 μm range, we posit that the device is more suited for thermal camouflage applications in the lower-temperature LWIR range.

To address the MWIR performance, we have developed an improved configuration, designated ‘SMDM-MWIR,’ which features enhanced suppression of MWIR emissions within the metasurface system. The optimal parameters for this configuration are: t_Ti_ = 120 nm, t_ZnS_ = 585 nm, t_Bi_ = 25 nm, D = 2400 nm, and P = 3300 nm. The spectral response of the ‘SMDM-MWIR’ configuration, shown in Figure S5 reveals an average emissivity of 0.39 (3–5 μm), 0.8 (5–8 μm), and 0.45 (8–14 μm), demonstrating a significant improvement in MWIR performance.

| **Radiation Ratio** | **300 K** | **350 K** | **400 K** | **450 K** | **500 K** |
| --- | --- | --- | --- | --- | --- |
| **3-5 μm** | 0.02 | 0.05 | 0.09 | 0.14 | 0.19 |
| **5-8 μm** | 0.25 | 0.31 | 0.36 | 0.39 | 0.4 |
| **8-14 μm** | 0.73 | 0.64 | 0.55 | 0.47 | 0.41 |

**Table S1.** The normalized radiance distribution of a blackbody at various temperatures across different waveband.


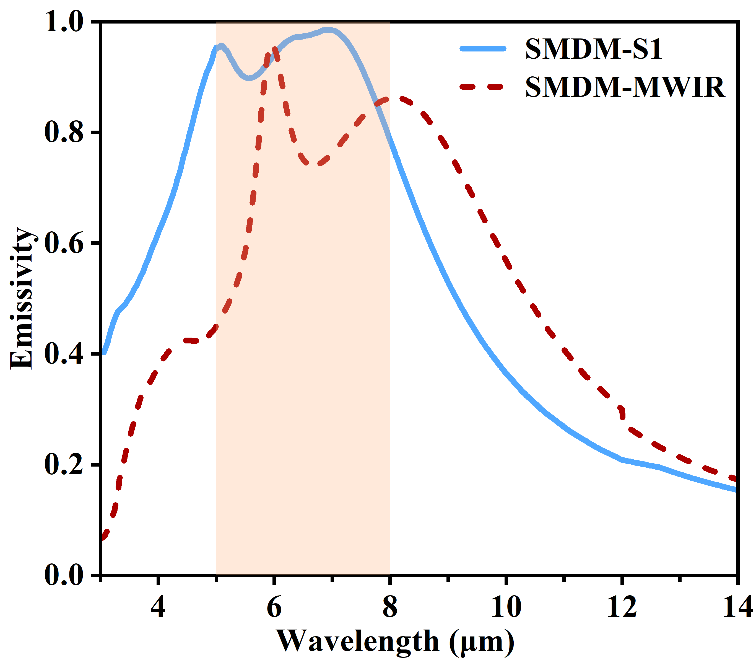


**Figure S5.** Simulated emission spectra for ‘SMDM-S1’, ‘SMDM-MWIR’.

Section S6 Effective metasurface impedance extraction

The equivalent impedance of a photonics system can be determined through the extraction of its *S*-parameters. Hence, we employed FDTD methods to extract the S-parameters of our SMDM-S1 metasurface. Subsequently, we derived its normalized equivalent impedance across the wavelength range of 3-14 μm utilizing the following equation.

$$Z_{eff}=\sqrt{\frac{\left( 1+S_{11} \right)^{2}-{S_{21}}^{2}}{\left( 1-S_{11} \right)^{2}-{S_{21}}^{2}}}$$

The three impedance rings (*A*=0.95, 0.9, 0.8), are represented by theirs trajectory equation about *R* and *L*, where *R* and *L* represent the real and imaginary parts of the impedance, expressed as the following equation.

$\left( 1-A \right)=\left| \frac{Z_{0}-\left( R+jL \right)}{Z_{0}+\left( R+jL \right)} \right|^{2}$

Section S7 Average emissivity simulation and experiment of the sample in non-ATW.

We conducted a comprehensive analysis to ascertain the average emissivity of the SMDM-S1 under non-polarized conditions within the non-ATW, considering incidence angles ranging from 0 to 70°. We involved computational simulations and FTIR measurement. The outcomes reveal that the sample consistently upholds an emissivity surpassing 0.8 across incidence angles spanning from 0 to 60°, thereby attesting to its commendable angular robustness.


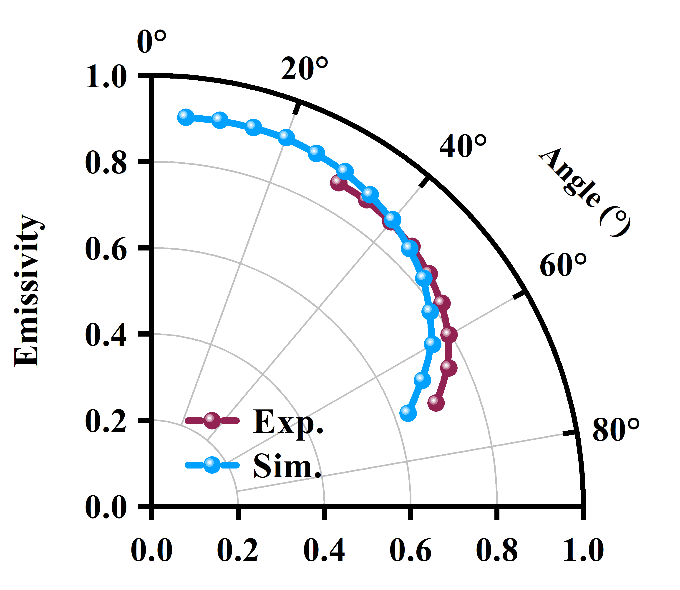


**Figure S7.** Average emissivity of the SMDM-S1 in non-ATW with different incident angle both experiment (red dots line) and simulation (blue dots line).

Section S8 Spectral radiance, atmospheric radiance power, solar radiance power, average emissivity calculation and reference sample design.

The formula that we use to calculate the spectral radiation intensity of our ‘SMDM-S1’ and reference sample as below:

$$P_{rad}\left( \lambda,T \right)=\varepsilon_{s}\left( \lambda,T \right)\cdot M_{bb}(\lambda,T)$$

Here, *ε_s_*(𝜆) represents the emissivity of the sample, which can be obtained from the measured spectrum, and 𝑀_bb_(𝜆, 𝑇) denotes the blackbody radiation as described by Planck’s Law.

To further understand the real-world applications of our ‘SMDM-S1’ sample by considering atmospheric and solar radiation in the 3-14 μm range. To address this, we have conducted calculations using the following formulas to determine the the atmospheric radiation power $P_{ats}$ and solar radiation power $P_{sol}$ [1]:

$$P_{ats}=\int_{\lambda_{1}}^{\lambda_{2}} \varepsilon_{\mathrm{ats}}\left( \lambda\right)\cdot M_{bb}\left( \lambda,T_{ats} \right)\cdot\varepsilon_{s}(\lambda)d\lambda$$

$$P_{sol}=\int_{\lambda_{1}}^{\lambda_{2}} \varepsilon_{s}\left( \lambda\right)\cdot I_{AM1.5}(\lambda)d\lambda$$

Here, *ε*_ats_(𝜆), *I_AM1.5_(λ)* indicates atmospheric emissivity spectrum and solar irradiance spectrum, respectively[2].

We have conducted detailed calculations to assess the external radiation contributions from the atmosphere and solar light to our ‘SMDM-S1’ sample. The results indicate that the total external radiation contribution to our ‘SMDM-S1’ sample is 50.38 W/m². For further context, we refer to Ref. 1 and Ref. 2, which provide calculated values of 17.34 W/m² and 7.6 W/m², respectively, for atmospheric and solar radiation. Despite our SMDM sample absorbing a higher amount of atmospheric and solar radiation, its unique ability to dissipate passive heat effectively in the 5-8 μm range enables it to maintain lower actual temperatures compared to the reference samples. This characteristic is particularly significant for applications where temperature management is crucial. The Ref.1 designed here is a multilayer planar structure composed of Ti, ZnS and Bi. The average emissivity from $\lambda_{1}$ to $\lambda_{2}$ can be calculated using the following formula:

$$\bar{\varepsilon}_{\lambda_{1}-\lambda_{2}}=\frac{\int_{\lambda_{1}}^{\lambda_{2}} \varepsilon_{s}(\lambda)\cdot M_{bb}\left( \lambda, T \right) d\lambda}{\int_{\lambda_{1}}^{\lambda_{2}} M_{bb}\left( \lambda, T \right) d\lambda}$$

Consequently, the average emissivity is dependent on temperature.

At room temperature, the average emissivity of the metasurface, calculated from the measured spectrum, is 0.72 (3~5 µm), 0.91 (5~8 µm), and 0.33 (8~14 µm). Subsequently, Ref. 1, consisting of Bi (153 nm), ZnS (400 nm), and Ti (120 nm) layers arranged from top to bottom, was designed using a parameter scanning method to confirm the average emissivity near the ‘SMDM-S1’ in the LWIR range. This structure was then fabricated surrounding the ‘SMDM-S1’. The average emissivity calculated from the measured spectrum of the Ref.1 is 0.21 (3~5 µm), 0.14 (5~8 µm), and 0.35 (8~14 µm). Additionally, the average emissivity of Ref.2 (Ti 120 nm, serving as the metal reference, is 0.16 (3~5 µm), 0.11 (5~8 µm), and 0.09 (8~14 µm). For infrared image measurements using an LWIR infrared camera operating in the 8~14 µm, the emissivity of the sample, the reference sample, and the metal are 0.343, 0.338, and 0.088 at 135℃, respectively. The corresponding spectra, measured before and after heating on a hotplate, are illustrated in Figure S8(a). Furthermore, we calculated the emission power of the reference sample and the metal over different temperatures in the 3–14 µm range, as shown in Figure S8(b). The external emission power at different temperatures (300 K, 400 K, 500 K) in non-attenuated total reflection (non-ATR) conditions is 6.45 W/m², 41.15 W/m², and 129.57 W/m² for the Ref.2, and 7.91 W/m², 48.62 W/m², and 149.82 W/m² for the Ref.1.


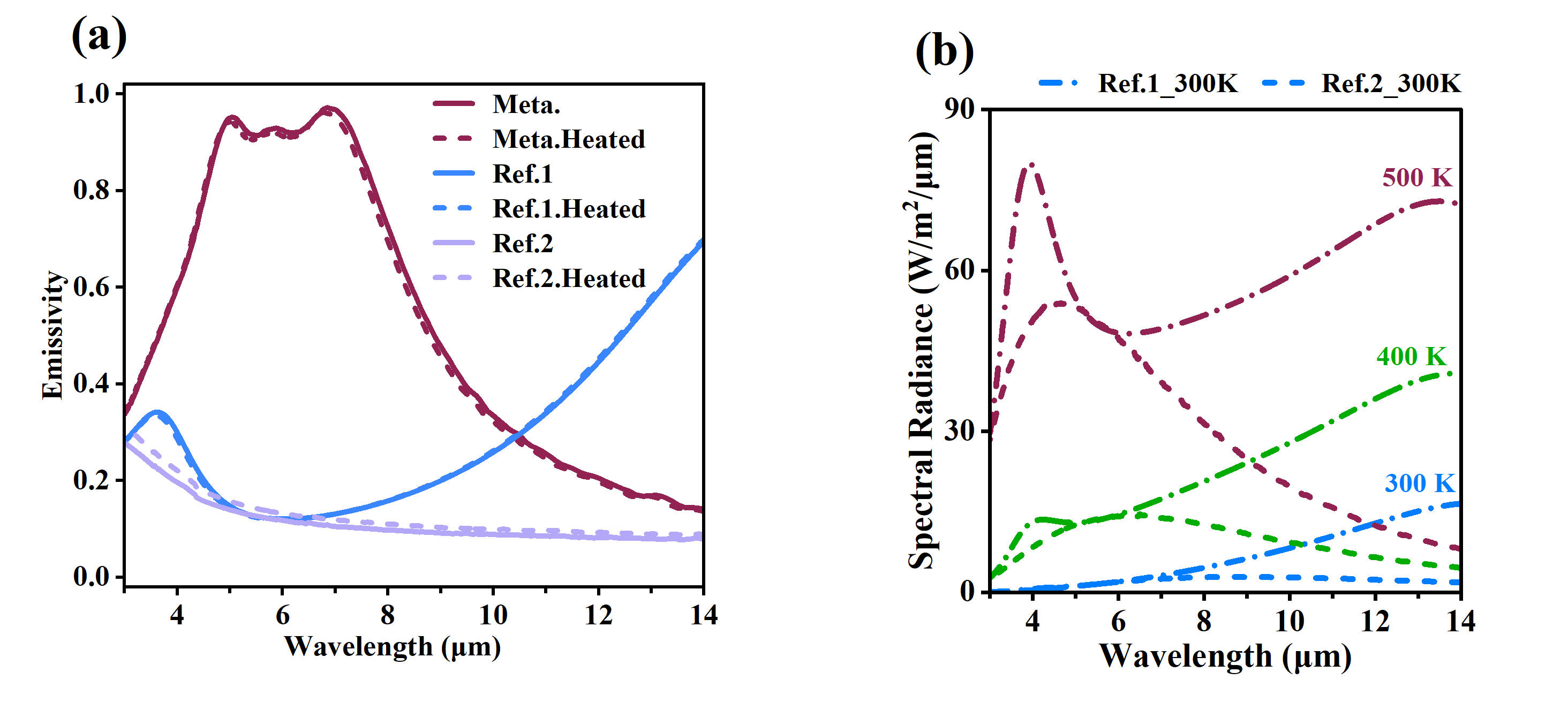


**Figure S8**. (a) Measured emissivity spectra of metasurface, Ref.1, and Ref.2 before and after heated to about 135 ̊C. (b) Spectral radiation of Ref.1 and Ref.2 at various temperatures.

Section S9 A photograph of experimental setup for thermal management.


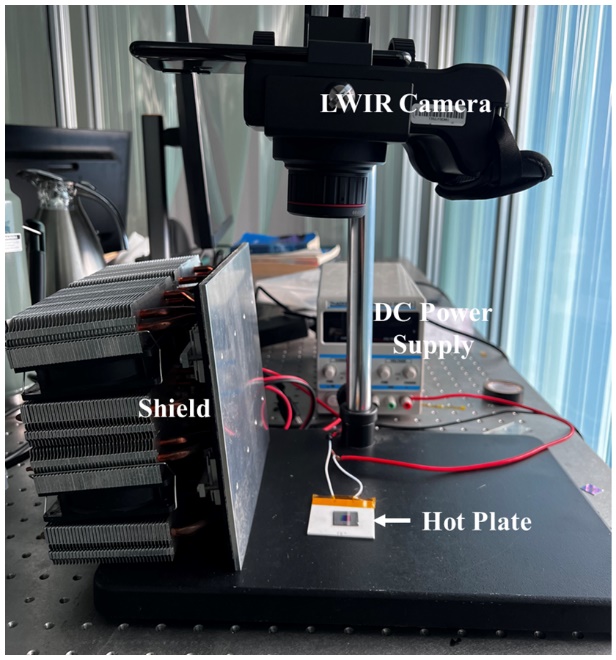


**Figure S9.** (a) The visible photograph of the experimental scene.

Section S10 An infrared Image captured by the infrared camera with the emissivity of E=0.09


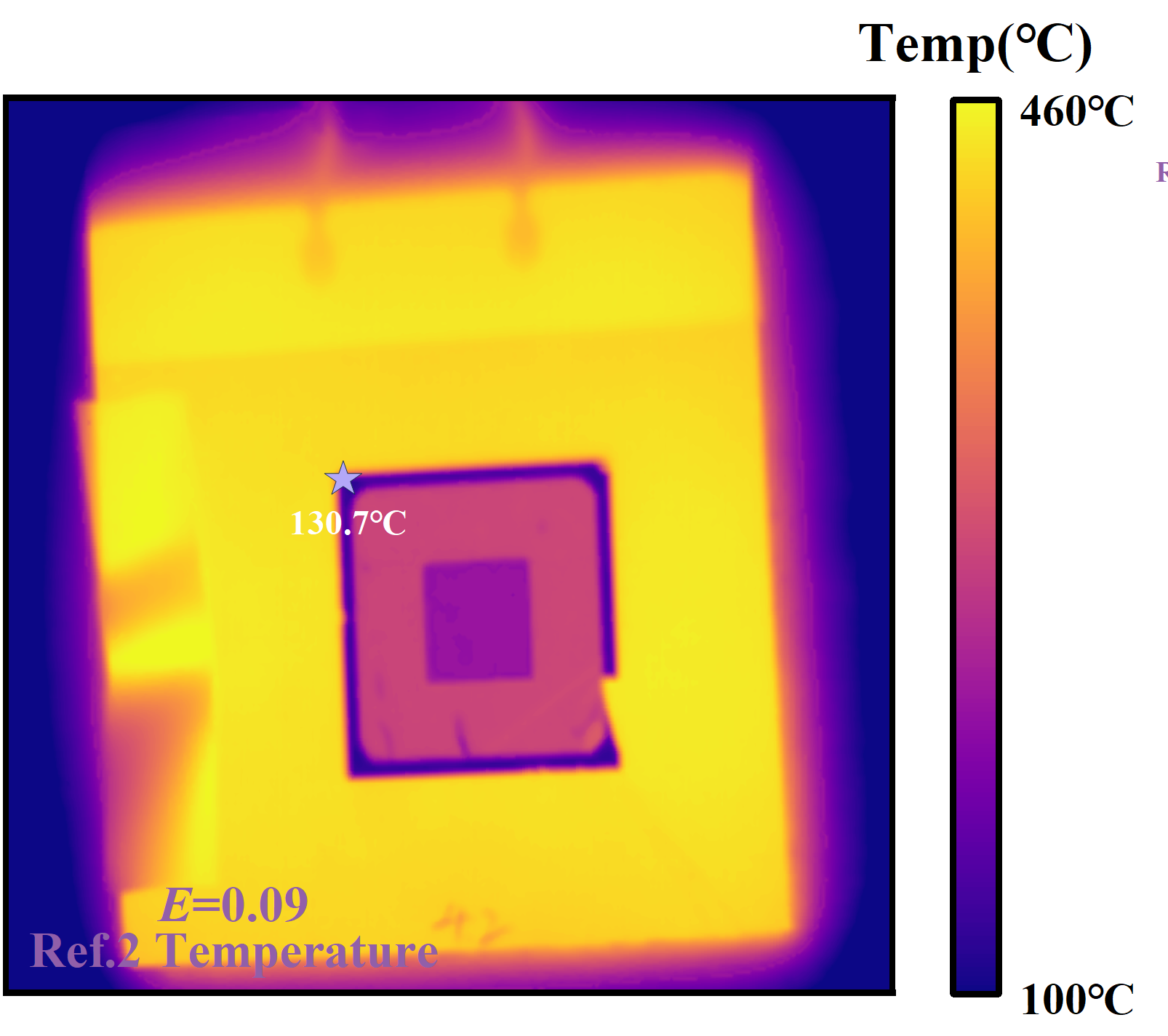


**Figure S10.** (a) LWIR image for the in-camera emissivity setting as 0.09.

Section S11 Numerical calculation of temperature difference between metasurface, Ref.1, and Ref.2.

In Figure 5, we observe the temporal evolution of both the radiation and actual temperatures for the metasurface, Ref.1, and Ref.2, each subjected to a constant heating power of 2400 W/m². At steady state, the radiation temperatures of the metasurface, Ref.1, and Ref.2 were recorded as 55.4°C, 69.7°C, and 48.2°C, respectively, as illustrated in Figure 5(d). These measurements were obtained using a LWIR camera with a surface emissivity (*ε*) set to 0.95. Notably, the actual temperature of the hotplate was approximately 135°C. By adjusting the in-camera emissivity setting to 0.34, as calculated in Section 6, the actual temperatures of the metasurface and Ref.1 were determined to be 106.0°C and 128.2°C, respectively. Additionally, the actual temperature of the Ref.2 was found to be 130.7°C by setting the in-camera emissivity to 0.09.

Subsequently, the numerical calculation of the temperature difference at steady state was performed using the Finite Element Method (FEM). the metasurface, Ref.1, and Ref.2 were modeled as nested silicon cubes. The evaporation layer was excluded from the heat transfer simulations, given that its heat capacity is negligible compared to that of the silicon substrate. The geometrical parameters were set to match those of the fabricated samples, with the metasurface region dimensions being a 1.1 cm (length) × 1.1 cm (width) × 0.5 cm (height) cube, Ref.1 comprising a square-hole ring with an outer diameter of 1.8 cm, an inner diameter of 1.1 cm, and a height of 0.5 cm, and Ref.2 being a also square-hole ring with an outer diameter of 2.0 cm, an inner diameter of 1.8 cm, and a height of 0.5 cm.

Natural convective heat flux boundary conditions were applied to both the top and side surfaces of the cubes. Additionally, to account for thermal radiation, surface-to-ambient radiation conditions were applied to the top surfaces of all samples. The emissivity values for these top surfaces were set to the average emissivity of the metasurface, Ref.1, and Ref.2 at 135°C, calculated from measured spectra in the range of 3 to 14 µm, resulting in values of 0.61, 0.24, and 0.11, respectively. The bottom surfaces were subjected to general inward heat flux boundary conditions.

In the simulation, the heat flux was set to 2350 W/m², slightly lower than the actual heating power (2400 W/m²) of the hotplate, due to imperfect thermal contact between the sample and the hotplate. The results obtained from the steady-state simulation revealed temperatures of 105.2°C (SMDM-S1), 126.8°C (Ref.1), and 130.7°C (Ref.2).
